# Position-specific sagittal-plane right knee kinematics during spike take-off in male volleyball players

**DOI:** 10.3389/fphys.2026.1931891

**Published:** 2026-09-18

**Authors:** Huiteng Xu, Shouhang Li, Yeon-hee Seo

**Affiliations:** 1 Department of Physical Education, Woosuk University, Wanju-gun, Jeollabuk-do, Republic of Korea; 2 Physical Education Program, Graduate School, Shandong Sport University, Jinan, China; 3 College of Agriculture and Forestry Engineering, Xuzhou Vocational College of Bioengineering, Xuzhou, China

**Keywords:** playing position, right knee joint, sagittal-plane kinematics, spike take-off, three-dimensional motion capture, volleyball

## Abstract

This study compared sagittal-plane right knee kinematics during position-specific spike take-off tasks among male volleyball outside hitters (OH), middle blockers (MB), and opposite hitters (OP). Twenty-seven male volleyball players classified as national first-level athletes were recruited and divided into three groups according to playing position, with nine athletes in each group. A three-dimensional motion capture system was used to collect right knee kinematic data. The instant of right-foot contact during the final approach step, the instant of minimum right knee angle, and the instant of bilateral take-off were defined as T1, T2, and T3, respectively. Right knee angle at key events, as well as the duration, range of motion, and phase-average angular velocity during the braking, propulsive, and total take-off phases, were analyzed. All three groups showed a similar kinematic pattern, with the right knee angle decreasing from T1 to T2 and increasing from T2 to T3. MB players showed significantly larger right knee angles at T1 and T2 than OH and OP players (162.04 ± 3.33° and 106.69 ± 8.02°, respectively; p < 0.001). OP players had the longest propulsive and total take-off durations, whereas MB players had the shortest durations (p < 0.001). Propulsive range of motion differed significantly among groups (Holm-adjusted p = 0.004), with OP players showing the greatest value (72.80 ± 9.30°) and larger values than OH and MB players. Total range of motion remained significant in the primary analysis (Holm-adjusted p = 0.030), with OP players showing larger values than MB players; however, this finding was not confirmed by the Holm-adjusted supplementary Kruskal–Wallis analysis and should therefore be interpreted cautiously. These findings characterize between-group differences in sagittal-plane right-knee kinematics under position-specific spike take-off task conditions. MB players showed larger right-knee angles and shorter take-off durations, OP players showed greater propulsive right-knee range of motion, and OH players showed intermediate values for several variables. These findings may help coaches identify right-knee kinematic variables to monitor within position-specific spike take-off training tasks.

## 1 Introduction

The volleyball spike is one of the most representative attacking techniques in competitive volleyball, and the approach take-off is a key phase for executing a high-quality spike (Oliveira et al., 2020; Fuchs et al., 2019). An athlete’s ability to complete an effective approach, braking action, propulsion, and take-off within a short time not only affects hitting height, hitting timing, and attacking effectiveness, but also reflects the coordinated control capacity of the lower-limb joints during rapid movement transitions (Tai et al., 2021; Pleša et al., 2021). During spike take-off, the lower limbs must lower the body’s center of mass, absorb impact, and adjust body posture after foot contact, before generating rapid upward displacement of the center of mass through the subsequent propulsive action (Bisseling et al., 2008). Therefore, the kinematic characteristics of the take-off phase provide an important perspective for understanding volleyball-specific movement performance, lower-limb functional control, and neuromuscular regulation (Oliveira et al., 2020; Tai et al., 2021).

As an important intermediate joint connecting the hip and ankle, the knee joint plays multiple roles in braking, support, and extension force generation during the approach take-off (Obara et al., 2022). In the sagittal plane in particular, knee flexion and extension directly contribute to braking regulation after foot contact and to propulsive transition before take-off (Bisseling et al., 2008; Obara et al., 2022). Knee joint angle reflects lower-limb posture at key events, temporal structure reflects the rhythm of the braking and propulsive phases, and joint range of motion and phase-average angular velocity further reveal the movement range and transition speed of the joint (Tai et al., 2021; Bisseling et al., 2008; Obara et al., 2022). Therefore, analyzing sagittal-plane knee angle, phase duration, range of motion, and phase-average angular velocity can characterize the timing and magnitude of right-knee flexion–extension during the braking and propulsive phases of volleyball take-off.

Although the present study focuses on take-off, a spike jump is a continuous take-off–flight–landing sequence. Across jump-landing tasks, peripheral fatigue can alter lower-limb landing kinematics and kinetics, although the effects vary across fatigue protocols (He et al., 2025b). Unanticipated sport-specific conditions have been associated with higher landing loading rates and knee extension moments, whereas chronic ankle instability has been linked to altered proximal-joint mechanics and greater vertical ground-reaction forces during landing (He et al., 2025a, 2024). This evidence provides a broader biomechanical context; however, only the take-off phase was measured, precluding direct inference about subsequent landing loads or injury risk.

Different playing positions in volleyball involve distinct position-specific task demands (Skazalski et al., 2018; Lima et al., 2019). Outside hitters, middle blockers, and opposite hitters differ in attacking zones, approach paths, take-off timing, attacking rhythm, and spike directions (Rebelo et al., 2025; Sheppard et al., 2009). Outside hitters typically perform powerful attacks from zone 4 and adjust their approach rhythm and hitting direction according to ball trajectory and defensive organization. Middle blockers often perform quick attacks close to the net, with a faster movement rhythm and greater demands on take-off timing and rapid transition ability. Opposite hitters frequently undertake attacks from zone 2 and back-row attacking tasks, which may involve different approach space, take-off preparation, and propulsive characteristics (Skazalski et al., 2018; Sheppard et al., 2009). These position-specific task demands may be associated with differences in sagittal-plane right-knee flexion–extension characteristics during take-off (Sattler et al., 2015).

Previous biomechanical studies in volleyball have mainly focused on spike performance, jump height, landing technique, lower-limb joint loading, and injury risk, providing an important basis for understanding lower-limb movement characteristics in volleyball players (Oliveira et al., 2020; Bisseling et al., 2008; Obara et al., 2022). However, several gaps remain. On the one hand, some studies have used standardized jumping tasks or general spike tasks, which may not fully preserve the position-specific attacking demands of different players (Tai et al., 2021; Lima et al., 2019). On the other hand, direct comparisons of sagittal-plane knee kinematics during the take-off phase among outside hitters, middle blockers, and opposite hitters performing position-specific spike tasks remain limited (Rebelo et al., 2025). In particular, further evidence is needed from analyses that integrate right knee angle, temporal structure, range of motion, and phase-average angular velocity across the continuous movement window from right-foot contact of the final approach step to maximum knee flexion and bilateral take-off.

Accordingly, this study used three-dimensional motion capture to compare sagittal-plane right knee kinematic characteristics during position-specific spike take-off tasks among male volleyball players classified as national first-level athletes. The analyzed variables included right knee angle at T1, T2, and T3, as well as the duration, range of motion, and phase-average angular velocity of the braking, propulsive, and total take-off phases. This study aimed to characterize between-group differences in sagittal-plane right knee kinematics within a common take-off-phase analysis framework. We hypothesized that outside hitters, middle blockers, and opposite hitters would differ in these right knee kinematic variables under their respective position-specific spike task conditions.

## 2 Materials and methods

### 2.1 Participants

A total of 27 male volleyball players classified as national first-level athletes in China were recruited for this study. All participants were adult male volleyball players. According to their playing positions, the athletes were divided into three groups: outside hitters (OH), middle blockers (MB), and opposite hitters (OP), with nine athletes in each group. The sample size was determined by the number of national first-level athletes who met the inclusion criteria and were able to complete all testing procedures during the data collection period. All participants were right-handed spikers and performed the spike using the right arm. The right knee was selected to standardize the analyzed limb and the take-off window beginning at right-foot contact across groups; its functional role was not assumed to be identical across tasks or to represent the complete bilateral take-off strategy.

Before testing, participants’ age, height, body mass, body mass index (BMI), and training experience were recorded. No statistically significant differences were detected among the three groups in these characteristics (all p > 0.05; **Table 1**).

**Table 1:** Demographic, anthropometric, and training characteristics of participants.

Variable	Outside hitter (n = 9)	Middle blocker (n = 9)	Opposite hitter (n = 9)	p
Age (years)	21.78 ± 1.39	21.33 ± 2.00	21.89 ± 2.47	0.825
Height (cm)	191 ± 5	193 ± 3	191 ± 4	0.440
Body mass (kg)	81.78 ± 7.90	85.44 ± 8.06	79.33 ± 8.44	0.295
Training experience (years)	7.89 ± 1.96	7.33 ± 1.00	7.56 ± 1.42	0.739
BMI (kg/m²)	22.44 ± 1.14	22.93 ± 1.60	21.78 ± 1.58	0.262

Values are presented as mean ± SD. BMI, body mass index. p values were calculated using one-way analysis of variance.

The inclusion criteria were as follows: (1) at least 5 years of systematic volleyball training experience; (2) weekly training time of no less than 16 h; (3) no acute or chronic injury affecting athletic performance within the previous 12 months; (4) no neurological disease affecting lower-limb motor function; and (5) BMI within the range of 18.5–25.0 kg/m². The exclusion criteria were as follows: (1) pain or other discomfort during testing; and (2) chronic disease or long-term medication use that could affect athletic performance.

All participants were instructed to avoid strenuous exercise and alcohol consumption within 24 h before testing and to complete their last meal at least 2 h before the formal test. Previous injury history was confirmed through questionnaires and interviews before testing. All participants provided written informed consent. This study was reviewed and approved by the Research Ethics Committee of Shandong Sport University (Approval No. 2026032) and was conducted in accordance with the principles of the Declaration of Helsinki.

### 2.2 Experimental design and spike take-off tasks

This study used a cross-sectional comparative design based on three-dimensional motion capture to compare sagittal-plane right knee flexion–extension characteristics during position-specific spike take-off among OH, MB, and OP players. The experimental tasks were designed with reference to the typical attacking patterns of different playing positions in competition, while preserving their position-specific approach rhythm, take-off style, and spike direction characteristics (Lima et al., 2019; Sheppard et al., 2009). During testing, the same setter performed all sets from a fixed position to reduce the influence of inter-setter variability on movement performance. However, set trajectory and height, approach velocity, horizontal approach distance, center-of-mass velocity, jump height, take-off velocity, spike reach, and ball-contact performance were not quantitatively recorded or analyzed. Therefore, using the same setter reduced inter-setter variability but did not standardize these task-performance variables across the three position-specific tasks.

Before formal testing, the setter was explicitly instructed to provide the prescribed set for each position-specific task. OH players received a high outside set to zone 4 and performed a powerful diagonal spike, with the valid target area located in zone 5 of the opponent’s court. MB players received a quick set close to the setter in zone 3 and performed a near-net quick attack according to their usual quick-attack rhythm, with valid target areas located in zones 2 and 4 of the opponent’s court. OP players started from zone 1, received a high set to zone 1, and performed a back-row diagonal attack according to their usual back-row attacking rhythm, with valid target areas located in zones 1 and 6 of the opponent’s court (**Figure 1**).

**Figure 1:**
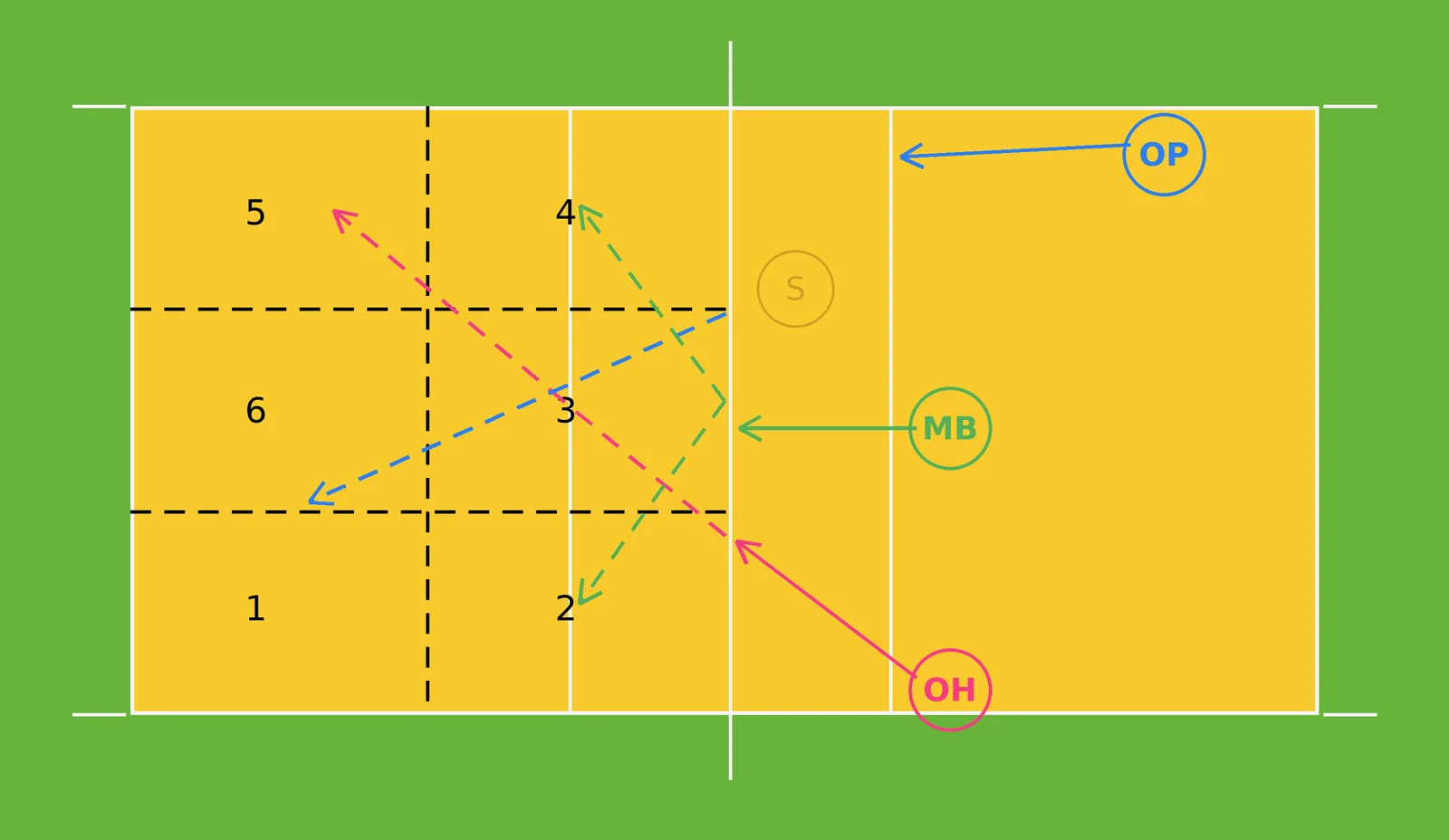
Schematic illustration of the position-specific spike take-off tasks for outside hitters, middle blockers, and opposite hitters. OH, outside hitter; MB, middle blocker; OP, opposite hitter; S, setter. Numbers 1–6 indicate volleyball court zones. Solid arrows indicate the players’ approach directions, whereas dashed arrows indicate spike directions and valid target areas for each playing position.

Because OH, MB, and OP players differ in approach step number, take-off timing, attacking rhythm, and spike direction as part of their position-specific demands (Skazalski et al., 2018; Sattler et al., 2015), the present study did not forcibly standardize the approach step number or spike rhythm across the three groups. Instead, these characteristics were retained as part of the position-specific spike task. To improve comparability across positions, the analysis window was standardized as the spike take-off phase from right-foot contact during the final approach step to bilateral take-off.

This study focused on the kinematic changes of the right knee joint during the take-off phase. T1 was defined as the instant of right-foot contact during the final approach step; T2 was defined as the instant at which the right knee angle reached its minimum value, representing the point of maximum right knee flexion during braking; and T3 was defined as the instant of bilateral take-off. Accordingly, T1–T2 was defined as the braking phase, T2–T3 as the propulsive phase, and T1–T3 as the total take-off phase. Subsequent analyses of right knee angle, duration, range of motion, and phase-average angular velocity were all based on this standardized movement window.

### 2.3 Testing procedure

Before formal testing, all participants completed a standardized 15-min warm-up, including jogging, dynamic stretching, and volleyball-specific preparatory activities. They then changed into standardized testing clothing and completed three familiarization spike trials.

Before data collection, spatial calibration of the motion capture system was performed. Subsequently, 47 reflective markers with a diameter of 14 mm were attached to the participants’ body surface according to the calibrated anatomical systems technique (CAST), and one static standing trial was collected to establish an individualized three-dimensional human body model (Cappozzo et al., 1995, 2005). During formal testing, each participant first passed the ball to the setter and then completed the approach, take-off, and spike according to the position-specific spike task.

Each participant completed six valid formal spike trials, with a 90-s interval between trials to reduce the influence of fatigue on movement performance. If a trial did not meet the preset criteria for set quality, movement continuity, marker completeness, or motion capture signal quality, it was considered invalid and repeated until six valid trials were obtained. Three national-level volleyball referees reviewed the six valid trials for each participant based on five qualitative criteria: approach quality, take-off quality, spike timing, movement fluency, and attacking effectiveness. The trial used for analysis was selected by consensus among the three referees. No numerical scoring scale was used, and independent referee ratings were not recorded; therefore, formal inter-rater agreement could not be calculated. Because the analysis focused on a technically successful position-specific spike execution rather than mean performance across repeated attempts, the six valid trials were not averaged. The trial judged by consensus to have the highest overall technical quality was used for analysis. Accordingly, this trial should be interpreted as a high-quality execution rather than as representative of each athlete’s typical or average performance; trial-to-trial reliability and within-player variability across the six trials could not be evaluated.

### 2.4 Data collection and processing

Three-dimensional kinematic data were collected using eight Qualisys A12 infrared high-speed cameras at a sampling frequency of 240 Hz (Cappozzo et al., 2005). Spatial calibration was performed before testing, and the calibration residual was maintained below 0.8 mm. The global coordinate system was defined as follows: the x-axis pointed toward the participant’s right sideline direction, the y-axis pointed in the direction of the approach, and the z-axis pointed vertically upward. After marker identification and trajectory inspection were completed in Qualisys Track Manager, the motion capture data were imported into Visual3D for three-dimensional body modeling and kinematic analysis (Leardini et al., 2005; Della Croce et al., 2005).

An individualized three-dimensional full-body model was established based on the CAST modeling method and the static standing trial (Cappozzo et al., 1995, 2005). Although a full-body marker set was used, the present analysis was specifically limited to sagittal-plane right knee flexion–extension kinematics. Hip, ankle, contralateral knee, and multi-joint coordination variables were not included in the present analysis. As illustrated in **Figure 2**, the right-knee angle was defined as the geometric included (internal) angle between the longitudinal axes of the right thigh and right shank, with the knee joint as the vertex. A smaller knee angle indicated greater knee flexion, whereas a larger knee angle indicated a knee position closer to extension (Grood and Suntay, 1983). Because this study focused on sagittal-plane right knee flexion–extension, frontal-plane and transverse-plane knee angles were not included in the analysis.

**Figure 2:**
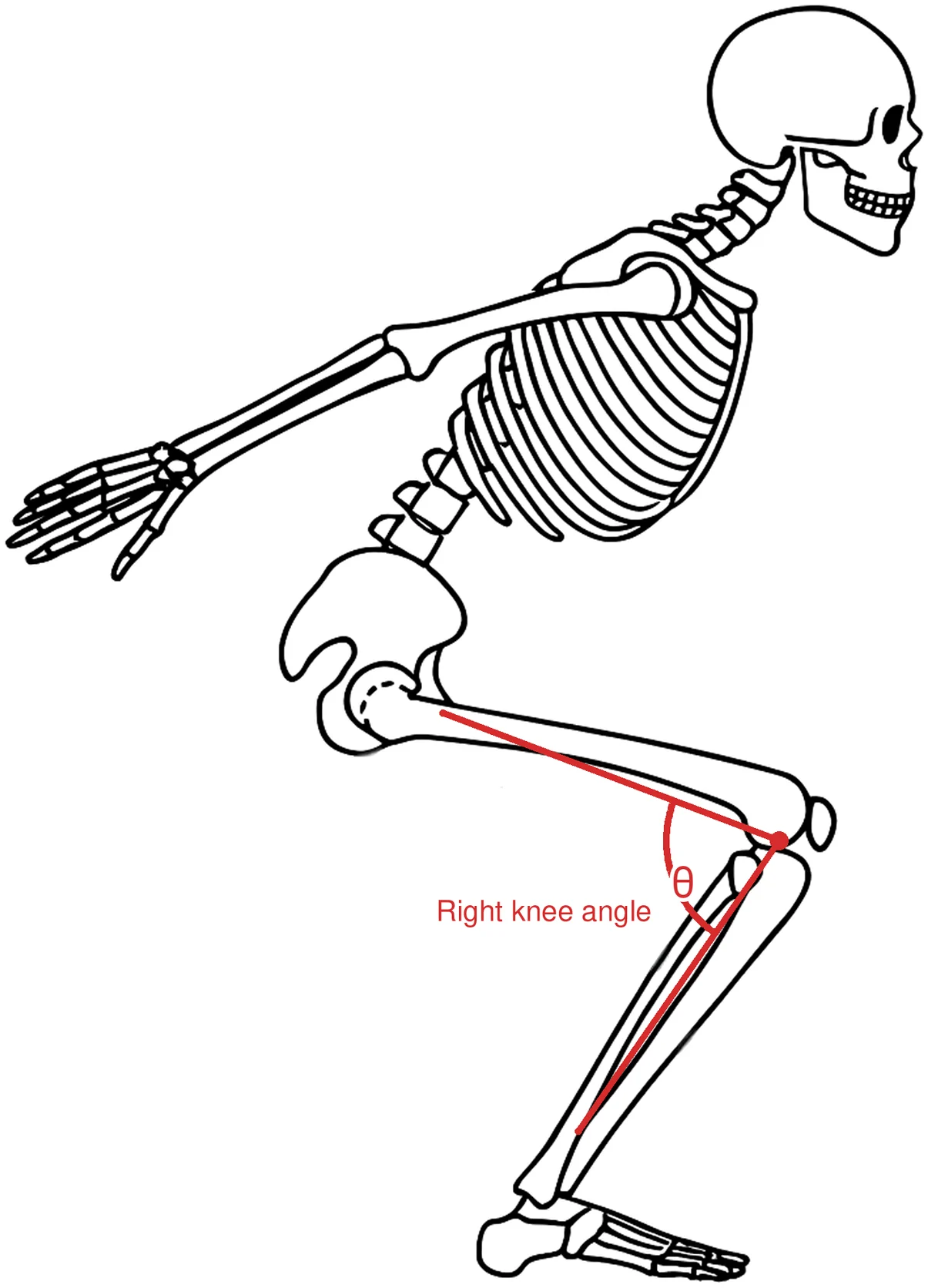
Schematic illustration of the sagittal-plane right-knee angle used in this study. The knee joint is the vertex, and θ denotes the geometric included (internal) angle between the longitudinal axes of the right thigh and right shank.

The raw right knee angle time series was smoothed using a 10-Hz Butterworth low-pass filter (Crenna et al., 2021; Kristianslund et al., 2012). T1 was identified as the first frame in which downward displacement of the right-foot markers ceased during final approach contact, and T3 as the first frame in which both feet showed continuous upward displacement after the final ground contact. Both events were determined from marker trajectories and frame-by-frame visual inspection. T2 was identified from the minimum value in the right knee angle time series. All events were identified by the same researcher; no fixed numerical threshold was applied, and intra-rater or inter-rater reliability was not assessed. The right knee angles at T1, T2, and T3 were then extracted, and the duration, range of motion, and phase-average angular velocity of the braking, propulsive, and total take-off phases were calculated.

Braking duration, propulsive duration, and total take-off duration were defined as the time intervals of T1–T2, T2–T3, and T1–T3, respectively. Braking range of motion was calculated as the absolute change in right knee angle between T1 and T2, whereas propulsive range of motion was calculated as the absolute change in right knee angle between T2 and T3. Total range of motion, representing total angular excursion during the entire take-off phase, was calculated as the sum of braking and propulsive range of motion. Braking phase-average angular velocity, propulsive phase-average angular velocity, and total phase-average angular velocity were calculated by dividing the corresponding angular displacement by the duration of each phase. Therefore, phase-average angular velocity in this study reflected the average magnitude of right knee angle change during the corresponding phase rather than directional angular velocity.

### 2.5 Statistical analysis

Primary statistical analyses were performed using IBM SPSS Statistics version 27.0; sensitivity and confidence-interval analyses were conducted in Python 3.13.5 using NumPy 2.3.5, SciPy 1.17.0, and statsmodels 0.14.6. Continuous variables are presented as mean ± standard deviation (SD). First, one-way analysis of variance (ANOVA) was used to compare age, height, body mass, BMI, and training experience among the OH, MB, and OP groups.

Before formal between-group comparisons, the Shapiro–Wilk test was used to assess the normality of each variable within each playing-position group, and Levene’s test was used to assess homogeneity of variance. With only nine participants per group, these tests had limited power; therefore, non-significant results were not considered definitive evidence that the distributional and variance assumptions were satisfied. One-way ANOVA or Welch’s ANOVA was used as the primary analysis. For variables satisfying the assumption of homogeneity of variance, one-way ANOVA was used to compare differences among the three groups. When a significant main effect was detected, Tukey’s *post hoc* test was used for pairwise comparisons. For variables violating the assumption of homogeneity of variance, Welch’s ANOVA was used, followed by Games–Howell *post hoc* comparisons. Considering the small sample size in each group and the presence of normality deviations in some variables, the Kruskal–Wallis test was additionally used as a supplementary non-parametric analysis to examine the robustness of the main findings. If a significant result in the primary analysis was not confirmed by the supplementary Kruskal–Wallis test, the interpretation of that result was treated with caution. Effect sizes for one-way ANOVA and Welch’s ANOVA were expressed as eta squared (η²), whereas effect sizes for the supplementary Kruskal–Wallis test were expressed as epsilon squared (ϵ²). For Holm-significant primary outcomes, 95% confidence intervals for η² were estimated using stratified bias-corrected and accelerated bootstrap resampling with 50,000 resamples, whereas pairwise confidence intervals were obtained from the corresponding Tukey or Games–Howell procedures. Complete statistical results are provided in **Supplementary Table S2**. To control the family-wise error rate across the 12 kinematic outcomes, Holm’s step-down correction was applied separately to the primary omnibus tests and the supplementary Kruskal–Wallis tests. *Post hoc* comparisons were interpreted only when the corresponding Holm-adjusted primary omnibus p value was < 0.05.

The main kinematic variables included right knee angle at T1, T2, and T3; braking duration, propulsive duration, and total take-off duration; braking range of motion, propulsive range of motion, and total range of motion; and braking phase-average angular velocity, propulsive phase-average angular velocity, and total phase-average angular velocity. MANOVA was not used because the small group sizes and exact linear dependencies among several outcomes would have made multivariate covariance estimation unstable. Each participant contributed one set of kinematic values derived from the consensus-selected high-quality trial. Therefore, the participant, rather than the individual trial, was the unit of analysis, with nine independent observations in each playing-position group. Because the sample size was based on athlete availability, no *a priori* power analysis was conducted; a sensitivity analysis for a three-group one-way ANOVA with N = 27, α = 0.05, and 80% power indicated a minimum detectable effect of Cohen’s f = 0.637, corresponding to η² = 0.288. All statistical tests were two-tailed, and a Holm-adjusted omnibus p value < 0.05 was considered statistically significant.

## 3 Results

Participant-level raw data are provided in **Supplementary Table S1**. Descriptive statistics, primary analysis results, effect sizes, *post hoc* comparisons, and supplementary Kruskal–Wallis test results for all kinematic variables, together with 95% confidence intervals for principal between-group differences and Holm-significant primary effect sizes, are presented in **Supplementary Table S2**.

### 3.1 Right knee joint angle changes

As shown in **Figure 3**, athletes in all three playing positions showed a similar right-knee flexion–extension pattern during spike take-off. Specifically, the right knee angle decreased from T1 to T2 as the knee flexed and then increased from T2 to T3 as the knee extended. A significant between-group difference was found in right knee angle at T1 (Holm-adjusted p < 0.001). *Post hoc* comparisons showed that MB players had a significantly larger right knee angle at T1 than both OH and OP players, indicating that the right knee was positioned closer to extension; no significant difference was observed between OH and OP players. The same between-group pattern was observed at T2: MB players showed a significantly larger right knee angle than both OH and OP players, indicating less knee flexion, whereas OH and OP players did not differ significantly from each other (Holm-adjusted p < 0.001). At T3, the between-group difference did not remain significant after Holm correction (F(2, 24) = 4.040, Holm-adjusted p = 0.123, η² = 0.252). The supplementary Kruskal–Wallis test was also non-significant after Holm correction (H(2) = 5.474, Holm-adjusted p = 0.259, ϵ² = 0.145).

**Figure 3:**
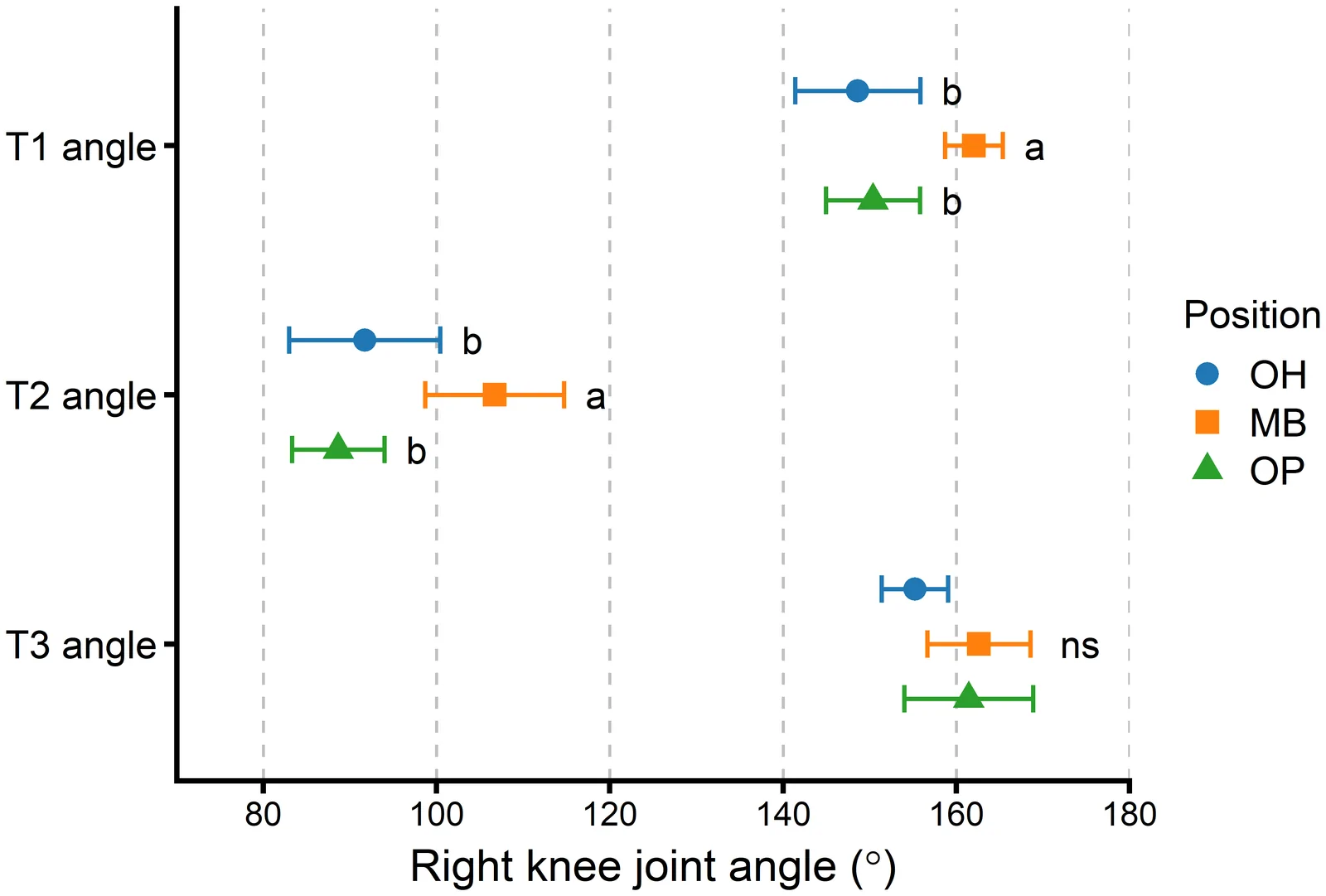
Comparison of right knee joint angles among playing positions. Data are presented as mean ± SD. OH, outside hitter; MB, middle blocker; OP, opposite hitter. T1 represents right-foot contact during the final approach step, T2 represents the instant of minimum right knee angle, and T3 represents bilateral take-off. Different lowercase letters indicate significant pairwise differences following a significant Holm-adjusted omnibus test; groups sharing the same letter are not significantly different. ns indicates no significant difference after Holm correction.

### 3.2 Temporal characteristics

As shown in **Figure 4**, the temporal characteristics of the right-knee braking, propulsive, and total take-off phases differed among the three groups under their respective task conditions. A significant between-group difference was found in braking duration (Holm-adjusted p = 0.014). *Post hoc* comparisons showed that OH players had a significantly longer braking duration than MB players, whereas OP players did not differ significantly from either OH or MB players.

**Figure 4:**
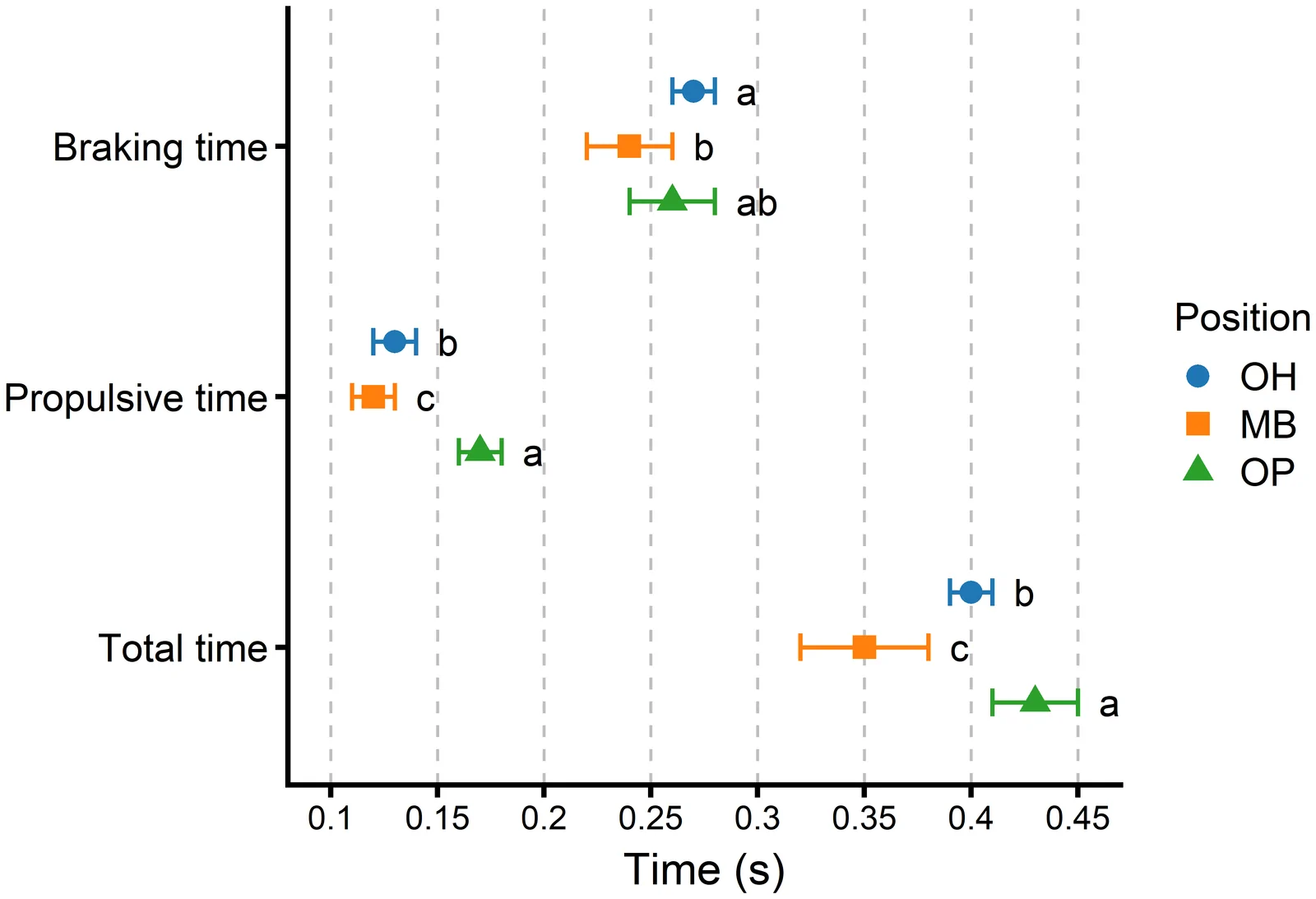
Comparison of right knee temporal variables among playing positions. Data are presented as mean ± SD. OH, outside hitter; MB, middle blocker; OP, opposite hitter. Braking duration is defined as the duration from T1 to T2, propulsive duration as the duration from T2 to T3, and total take-off duration as the duration from T1 to T3. Different lowercase letters indicate significant pairwise differences following a significant Holm-adjusted omnibus test; groups sharing the same letter are not significantly different.

Propulsive duration differed significantly among the three groups (Holm-adjusted p < 0.001). OP players showed the longest propulsive duration, followed by OH players, whereas MB players showed the shortest propulsive duration; all pairwise comparisons among the three groups were significant. Total take-off duration also showed a significant between-group difference (Holm-adjusted p < 0.001), with the same group ranking as propulsive duration: OP players had the longest total take-off duration, OH players showed intermediate values, and MB players had the shortest duration. All pairwise comparisons among the three groups were significant.

### 3.3 Right knee range of motion

As shown in **Figure 5**, right knee range of motion during the braking, propulsive, and total take-off phases showed different between-group patterns. No significant between-group difference was observed in braking range of motion after Holm correction (Holm-adjusted p = 0.400).

**Figure 5:**
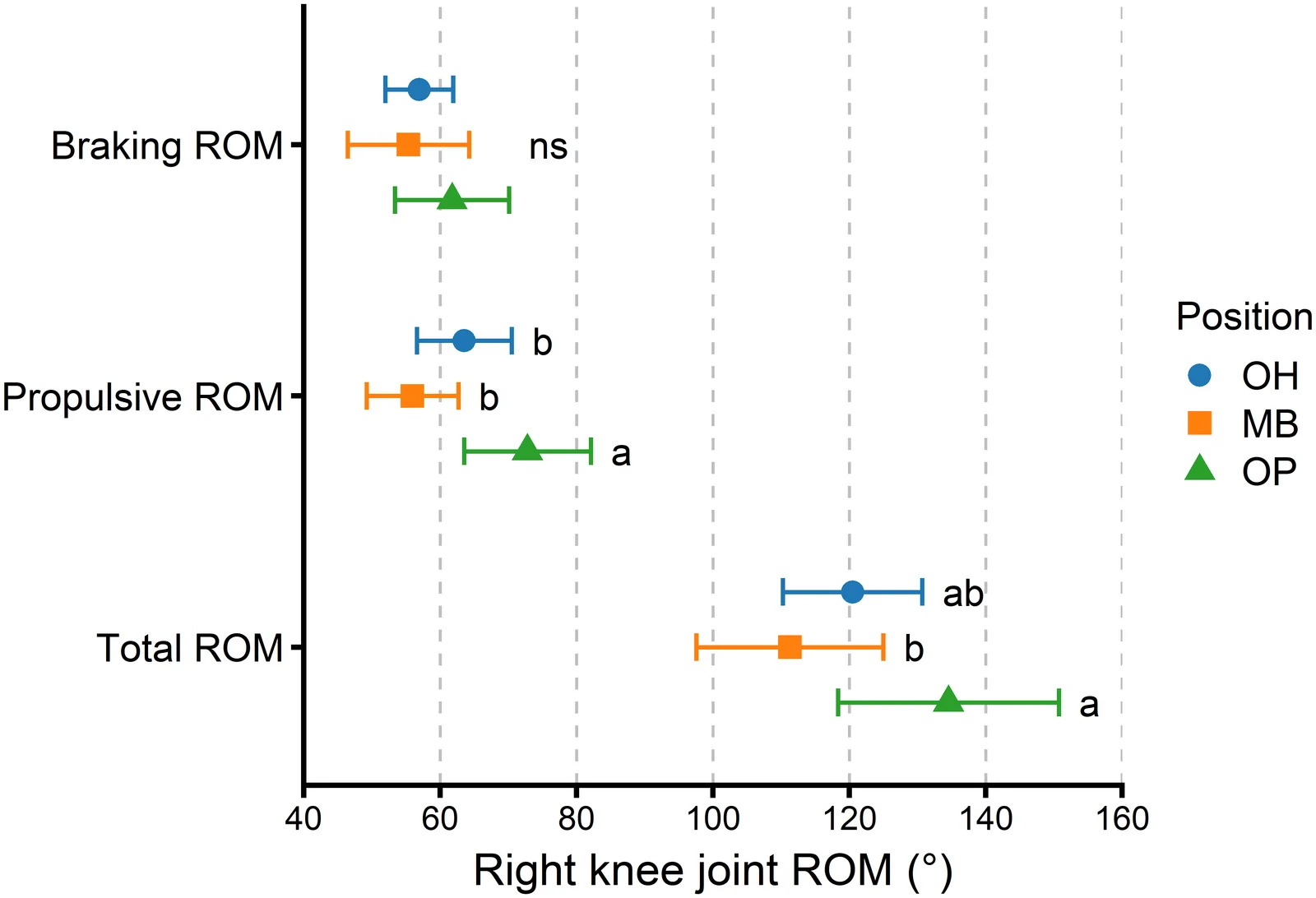
Comparison of right knee range of motion among playing positions. Data are presented as mean ± SD. OH, outside hitter; MB, middle blocker; OP, opposite hitter; ROM, range of motion. Braking ROM is defined as the angular change from T1 to T2, propulsive ROM as the angular change from T2 to T3, and total ROM as the total angular excursion during the entire take-off phase, calculated as the sum of braking and propulsive ROM. Different lowercase letters indicate significant pairwise differences following a significant Holm-adjusted omnibus test; groups sharing the same letter are not significantly different. ns indicates no significant difference after Holm correction.

During the propulsive phase, right knee range of motion differed significantly among the three groups (Holm-adjusted p = 0.004). *Post hoc* comparisons showed that OP players had a significantly greater propulsive range of motion than both OH and MB players, whereas no significant difference was observed between OH and MB players. Total range of motion remained significant in the primary analysis after Holm correction (Holm-adjusted p = 0.030). *Post hoc* comparisons indicated that OP players had a significantly greater total range of motion than MB players, whereas OH players showed intermediate values and did not differ significantly from either OP or MB players. However, this result was not confirmed by the Holm-adjusted supplementary Kruskal–Wallis test (Holm-adjusted p = 0.102) and should therefore be interpreted cautiously.

### 3.4 Right knee phase-average angular velocity

As shown in **Figure 6**, no significant between-group differences remained after Holm correction in braking, propulsive, or total take-off phase-average angular velocity (Holm-adjusted p = 0.122, 0.123, and 0.400, respectively). The Holm-adjusted supplementary Kruskal–Wallis tests were also non-significant for all three variables.

**Figure 6:**
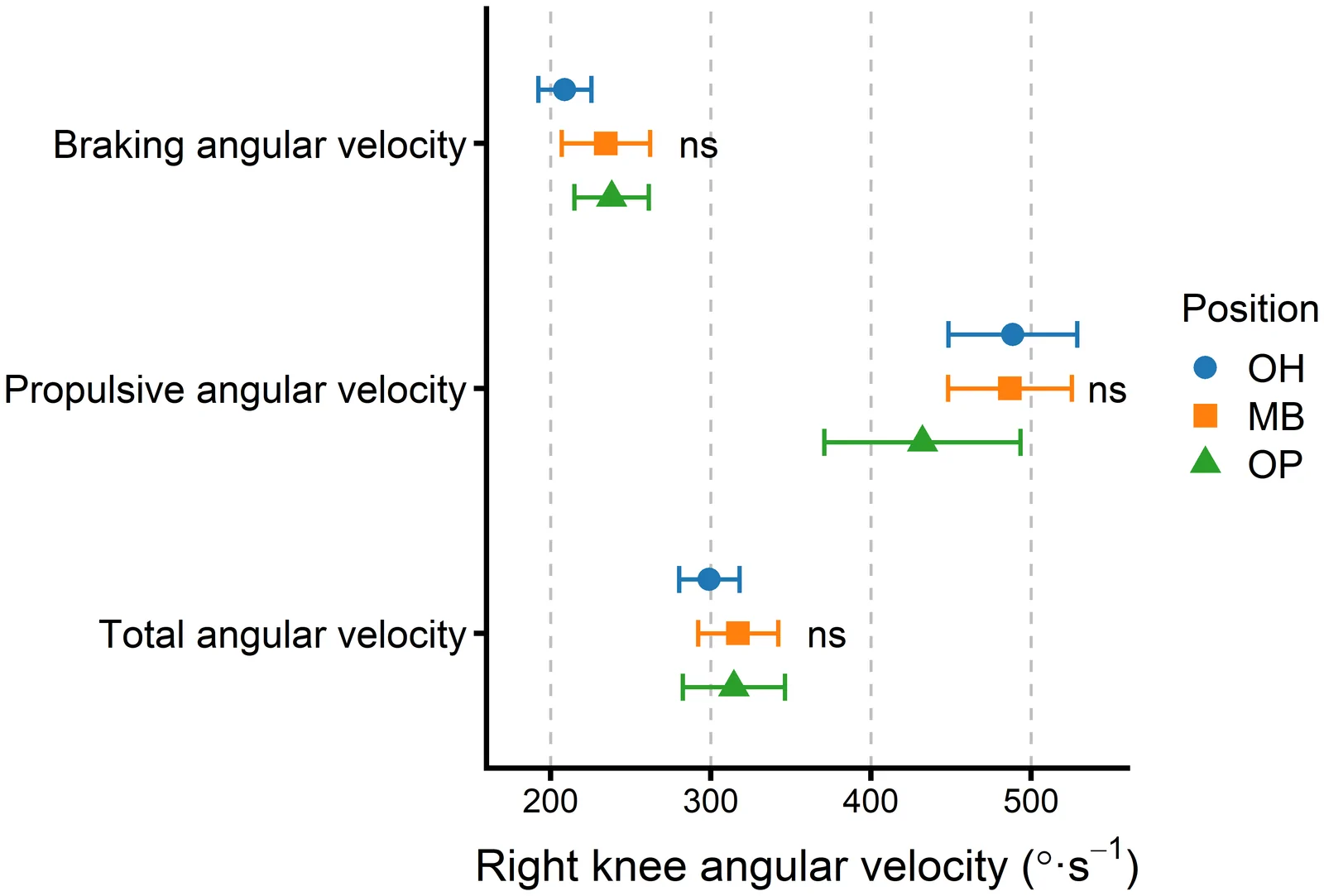
Comparison of right knee phase-average angular velocity among playing positions. Data are presented as mean ± SD. OH, outside hitter; MB, middle blocker; OP, opposite hitter. Braking phase-average angular velocity, propulsive phase-average angular velocity, and total phase-average angular velocity correspond to the T1–T2, T2–T3, and T1–T3 phases, respectively. Different lowercase letters indicate significant pairwise differences following a significant Holm-adjusted omnibus test; groups sharing the same letter are not significantly different. ns indicates no significant difference after Holm correction.

## 4 Discussion

This study compared right knee angle, temporal structure, range of motion, and phase-average angular velocity during position-specific spike take-off among male volleyball OH, MB, and OP players. The results showed that athletes in all three playing positions exhibited a similar right-knee flexion–extension pattern, with the knee flexing from T1 to T2 as the right knee angle decreased and extending from T2 to T3 as the angle increased. However, between-group differences were observed in several kinematic variables. MB players showed larger right knee angles at T1 and T2 and shorter take-off durations; OP players showed longer propulsive duration and greater propulsive range of motion; and OH players showed intermediate values for several variables. The total range-of-motion result should be interpreted cautiously. Although the three groups shared a similar right-knee flexion–extension sequence, several kinematic variables differed under the tested task conditions. Because each group performed a different spike task, these differences cannot be attributed to playing position independently of task-related mechanical effects.

### 4.1 Between-group differences in knee angle and temporal structure

The present study found that MB players had significantly larger right knee angles at both T1 and T2 than OH and OP players. This indicates that MB players maintained a relatively larger knee angle, and therefore a smaller degree of knee flexion, both at right-foot contact during the final approach step and at T2, the point of maximum right knee flexion during the braking phase. In addition, MB players showed shorter propulsive and total take-off durations. Taken together, under the tested quick-attack task condition, MB players showed larger right-knee angles and shorter propulsive and total take-off durations (Skazalski et al., 2018; Lima et al., 2019). Because jump height, take-off velocity, spike reach, and ball-contact performance were not quantified, the shorter take-off durations observed in MB players should be interpreted as temporal characteristics of the tested quick-attack task rather than as evidence of greater time efficiency or superior performance.

This pattern may be related to the position-specific attacking demands of middle blockers. MB players usually coordinate with the setter to perform quick attacks close to the net, which leaves a shorter preparation time for take-off and places high demands on attacking rhythm and take-off timing (Sattler et al., 2015). Therefore, the right-knee kinematic profile of MB players may be consistent with the shorter temporal demands of the quick-attack task. However, eccentric–concentric muscle function and dynamic stability were not directly measured in the present study (Sorenson et al., 2010; Markovic and Mikulic, 2010).

In contrast, OP players showed the longest propulsive and total take-off durations, indicating that they completed right-knee extension over a longer time interval. However, the effects of this temporal pattern on center-of-mass displacement, force generation, or lower-limb coordination cannot be determined from the present right-knee kinematic data. OH players showed intermediate values for several right-knee angle and temporal variables, but these findings alone do not establish a more balanced whole-body braking–propulsive strategy (Sheppard et al., 2009).

### 4.2 Range of motion during the braking and propulsive phases

This study found no significant between-group difference in braking range of motion, whereas propulsive range of motion remained significant after Holm correction. The total range-of-motion result should be interpreted cautiously because it was not confirmed by the corrected supplementary analysis. This suggests that the between-group differences observed under position-specific task conditions were concentrated in the propulsive phase rather than in the magnitude of knee flexion during braking (Bisseling et al., 2008; Sorenson et al., 2010).

OP players showed significantly greater propulsive range of motion than both OH and MB players. Their greater total range of motion than MB players was supported only by the corrected primary analysis. This indicates that OP players completed a larger right-knee angular excursion during the propulsive phase. However, the present analysis cannot determine how this greater excursion was coordinated with the hip and ankle or how it affected center-of-mass displacement and force generation (Yang et al., 2026). By contrast, although MB players maintained larger knee angles at T1 and T2, they did not show larger propulsive or total ranges of motion and completed take-off within a shorter time window. OH players showed intermediate values for range-of-motion variables under the tested task conditions. Overall, the observed between-group differences were mainly reflected in right-knee extension excursion and movement timing during the propulsive phase (Sattler et al., 2015).

### 4.3 Phase-average angular velocity during take-off

The phase-average angular velocity results showed no significant between-group differences after Holm correction. Although the three groups differed in braking duration, no phase-average angular velocity variable remained significant after correction. The supplementary Kruskal–Wallis tests were also non-significant after Holm correction. Nevertheless, braking-phase kinematics should be interpreted by jointly considering joint range of motion, movement duration, and phase-average angular velocity (Obara et al., 2022; Sorenson et al., 2010).

From the perspective of right-knee kinematics, a higher braking phase-average angular velocity indicates a greater magnitude of right-knee flexion per unit time during the braking phase; however, the associated muscular and stability demands cannot be determined from the present kinematic data. Accordingly, the descriptive angular-velocity differences observed among groups should not be interpreted as evidence of position-specific muscular demand, dynamic stability, or injury risk. Previous reviews have identified musculoskeletal injuries as important concerns in volleyball, and patellar tendinopathy may affect knee strength and jumping performance in professional volleyball players (Kilic et al., 2017; Chantrelle et al., 2022). Therefore, braking-control and lower-limb stability training may remain relevant in volleyball practice, but these recommendations are not directly supported by significant between-group angular-velocity differences in the present study (Silva et al., 2019; Ramirez-Campillo et al., 2020).

Propulsive phase-average angular velocity reached significance in the unadjusted primary analysis, but did not remain significant after Holm correction. Total take-off phase-average angular velocity also did not differ significantly among the three groups. Overall, phase-average angular velocity may serve as an auxiliary indicator for understanding speed regulation during take-off, whereas knee angle, temporal structure, and propulsive range of motion appear to better reflect the more robust between-group differences observed under the position-specific task conditions.

### 4.4 Implications for position-specific training

The present findings may help coaches identify right-knee kinematic variables to monitor within position-specific spike take-off training tasks. For MB players, coaches may monitor right knee angles at T1 and T2 and take-off duration during quick-attack drills, without interpreting shorter duration as a validated performance target. For OP players, propulsive duration and right knee range of motion may be monitored during back-row attack drills, with emphasis on maintaining technical control throughout the larger propulsive excursion (Yang et al., 2026; Ramirez-Campillo et al., 2021). For OH players, coaches may monitor right knee angle and phase timing during drills with varied approach rhythms and set conditions (Yang et al., 2026; Silva et al., 2019). These recommendations represent monitoring priorities rather than validated training prescriptions; the present data do not establish optimal target values, training loads, or causal training effects.

These results describe between-group differences in right-knee kinematics under the tested task conditions and do not establish that athletes in a particular position have a higher risk of knee injury (de Azevedo Sodré Silva et al., 2023; Zahradnik et al., 2014; Hewett et al., 2005; Leppänen et al., 2017). Knee injury risk is influenced by factors including ground reaction force, knee joint moment, knee valgus angle, muscle activation, previous injury, and training load (Paterno et al., 2010). Because these variables were not measured, any discussion of knee loading or injury risk should be regarded as a potential implication rather than direct evidence.

### 4.5 Limitations and future directions

This study has several limitations. First, each group included only nine athletes, and one consensus-selected high-quality trial per athlete was analyzed. Because independent referee ratings and repeated-trial kinematic summaries were unavailable, formal inter-rater agreement, trial-to-trial reliability, and the representativeness of the selected trial relative to typical performance could not be assessed. This may have underestimated within-player variability, introduced selection bias, and affected the magnitude of the observed between-group differences. The small sample provided sensitivity mainly for large effects; therefore, non-significant findings do not exclude smaller differences. In addition, the T3 angle and braking and propulsive phase-average angular-velocity results did not remain significant after Holm correction, and the total range-of-motion result was not confirmed by the Holm-adjusted supplementary analysis.

Second, event detection relied on marker trajectories and visual inspection without fixed numerical thresholds or formal reliability assessment, and the final right–left foot-contact sequence was not formally quantified. The analysis was limited to sagittal-plane right-knee kinematics and did not include the contralateral limb, multi-joint coordination, kinetics, electromyography, or landing. Accordingly, bilateral coordination, joint loading, subsequent landing loads, and injury risk cannot be inferred. Third, although the same setter delivered all sets from a fixed position to reduce inter-setter variability, the prescribed sets still differed qualitatively across tasks: OH players were instructed to receive a high outside set to zone 4, MB players a near-net quick set in zone 3, and OP players a high set to zone 1. However, the actual set height, trajectory, and timing relative to the athlete’s approach were not quantitatively recorded. These unmeasured set characteristics may have influenced approach initiation and the coordination of the braking and propulsive phases, potentially affecting knee angles, range of motion, phase durations, and phase-average angular velocity. Thus, the shorter propulsive duration observed in MB players than in OH players may partly reflect the different temporal demands of the quick set and high outside set. Because approach step number, rhythm, and take-off timing were also not standardized, the findings should be interpreted as differences under position-specific task conditions rather than as independent playing-position effects.

Future studies should use larger samples, analyze multiple trials, apply objective event-detection procedures with reliability assessment, and combine bilateral motion capture with force platforms and electromyography across take-off and landing. Intervention studies are also needed to determine whether the proposed monitoring priorities improve take-off kinematics and to establish appropriate target values and training doses.

## 5 Conclusion

Across all three groups, the right knee flexed from T1 to T2 and extended from T2 to T3, although several kinematic variables differed under the tested task conditions. MB players had larger right knee angles at T1 and T2 and a shorter total take-off duration, whereas OP players had a longer propulsive duration and greater propulsive range of motion; OH players showed intermediate values for several variables. These results are limited to sagittal-plane right-knee kinematics and do not represent complete lower-limb or multi-joint coordination. Practically, coaches may use these observed kinematic profiles to monitor take-off duration during MB quick-attack drills, technical control throughout the propulsive excursion during OP back-row attack drills, and right-knee angle and phase timing across varied approach rhythms during OH drills. These applications should be regarded as monitoring priorities rather than validated target values or training prescriptions.

## Data Availability

The original contributions presented in the study are included in the article/**Supplementary Material**. Further inquiries can be directed to the corresponding author.

## References

[B1] Bisseling R. W. Hof A. L. Bredeweg S. W. Zwerver J. Mulder T. (2008). Are the take-off and landing phase dynamics of the volleyball spike jump related to patellar tendinopathy? Br. J. Sports. Med. 42, 483–489. doi: 10.1136/bjsm.2007.044057 18385187

[B2] Cappozzo A. Catani F. Della Croce U. Leardini A. (1995). Position and orientation in space of bones during movement: anatomical frame definition and determination. Clin. Biomech. 10, 171–178. doi: 10.1016/0268-0033(95)91394-T 11415549

[B3] Cappozzo A. Della Croce U. Leardini A. Chiari L. (2005). Human movement analysis using stereophotogrammetry. Part 1: theoretical background. Gait. Posture. 21, 186–196. doi: 10.1016/j.gaitpost.2004.01.010 15639398

[B4] Chantrelle M. Menu P. Gernigon M. Louguet B. Dauty M. Fouasson-Chailloux A. (2022). Consequences of patellar tendinopathy on isokinetic knee strength and jumps in professional volleyball players. Sensors 22, 3590. doi: 10.3390/s22093590 35591280 PMC9105239

[B5] Crenna F. Rossi G. B. Berardengo M. Antonaci F. (2021). Filtering biomechanical signals in movement analysis. Sensors 21, 4580. doi: 10.3390/s21134580 34283131 PMC8271607

[B6] de Azevedo Sodré Silva A. Sassi L. B. Martins T. B. de Menezes F. S. Migliorini F. Maffulli N. . (2023). Epidemiology of injuries in young volleyball athletes: a systematic review. J. Orthop. Surg. Res. 18, 748. doi: 10.1186/s13018-023-04224-3 37789463 PMC10548731

[B7] Della Croce U. Leardini A. Chiari L. Cappozzo A. (2005). Human movement analysis using stereophotogrammetry. Part 4: assessment of anatomical landmark misplacement and its effects on joint kinematics. Gait. Posture. 21, 226–237. doi: 10.1016/j.gaitpost.2004.05.003 15639401

[B8] Fuchs P. X. Menzel H. J. K. Guidotti F. Bell J. von Duvillard S. P. Wagner H. (2019). Spike jump biomechanics in male versus female elite volleyball players. J. Sports. Sci. 37, 2411–2419. doi: 10.1080/02640414.2019.1639437 31280702

[B9] Grood E. S. Suntay W. J. (1983). A joint coordinate system for the clinical description of three-dimensional motions: application to the knee. J. Biomech. Eng. 105, 136–144. doi: 10.1115/1.3138397 6865355

[B10] He Z. Fang Z. Ye H. Su S. (2025a). Impact of unanticipated and backhand area smash landing on the lower limb biomechanics of female badminton players. Front. Bioeng. Biotechnol. 13, 1609911. doi: 10.3389/fbioe.2025.1609911 40521101 PMC12162505

[B11] He Z. Sun G. Zhu H. Ye B. Zheng Z. He X. . (2025b). Effects of different peripheral fatigue protocol on lower limb biomechanical changes during landing and its impact on the risk of anterior cruciate ligament injury: a systematic review. Front. Bioeng. Biotechnol. 13, 1587573. doi: 10.3389/fbioe.2025.1587573 40567252 PMC12188353

[B12] He Z. Zhu H. Ye B. Zheng Z. Liu G. Pan H. . (2024). Does chronic ankle instability patients lead to changes in biomechanical parameters associated with anterior cruciate ligament injury during landing? A systematic review and meta-analysis. Front. Physiol. 15, 1428879. doi: 10.3389/fphys.2024.1428879 39268191 PMC11391935

[B13] Hewett T. E. Myer G. D. Ford K. R. Heidt R. S. Colosimo A. J. McLean S. G. . (2005). Biomechanical measures of neuromuscular control and valgus loading of the knee predict anterior cruciate ligament injury risk in female athletes: a prospective study. Am. J. Sports. Med. 33, 492–501. doi: 10.1177/0363546504269591 15722287

[B14] Kilic O. Maas M. Verhagen E. Zwerver J. Gouttebarge V. (2017). Incidence, aetiology and prevention of musculoskeletal injuries in volleyball: a systematic review of the literature. Eur. J. Sport. Sci. 17, 765–793. doi: 10.1080/17461391.2017.1306114 28391750

[B15] Kristianslund E. Krosshaug T. van den Bogert A. J. (2012). Effect of low pass filtering on joint moments from inverse dynamics: implications for injury prevention. J. Biomech. 45, 666–671. doi: 10.1016/j.jbiomech.2011.12.011 22227316

[B16] Leardini A. Chiari L. Della Croce U. Cappozzo A. (2005). Human movement analysis using stereophotogrammetry. Part 3: soft tissue artifact assessment and compensation. Gait. Posture. 21, 212–225. doi: 10.1016/j.gaitpost.2004.05.002 15639400

[B17] Leppänen M. Pasanen K. Kujala U. M. Vasankari T. Kannus P. Äyrämö S. . (2017). Stiff landings are associated with increased ACL injury risk in young female basketball and floorball players. Am. J. Sports. Med. 45, 386–393. doi: 10.1177/0363546516665810 27637264

[B18] Lima R. F. Palao J. M. Clemente F. M. (2019). Jump performance during official matches in elite volleyball players: a pilot study. J. Hum. Kinet. 67, 259–269. doi: 10.2478/hukin-2018-0080 31523323 PMC6714353

[B19] Markovic G. Mikulic P. (2010). Neuro-musculoskeletal and performance adaptations to lower-extremity plyometric training. Sports. Med. 40, 859–895. doi: 10.2165/11318370-000000000-00000 20836583

[B20] Obara K. Chiba R. Takahashi M. Matsuno T. Takakusaki K. (2022). Knee dynamics during take-off and landing in spike jumps performed by volleyball players with patellar tendinopathy. J. Phys. Ther. Sci. 34, 103–109. doi: 10.1589/jpts.34.103 35221512 PMC8860694

[B21] Oliveira L. S. Moura T. B. M. A. Rodacki A. L. F. Tilp M. Okazaki V. H. A. (2020). A systematic review of volleyball spike kinematics: implications for practice and research. Int. J. Sports. Sci. Coach. 15, 239–255. doi: 10.1177/1747954119899881

[B22] Paterno M. V. Schmitt L. C. Ford K. R. Rauh M. J. Myer G. D. Huang B. . (2010). Biomechanical measures during landing and postural stability predict second anterior cruciate ligament injury after anterior cruciate ligament reconstruction and return to sport. Am. J. Sports. Med. 38, 1968–1978. doi: 10.1177/0363546510376053 20702858 PMC4920967

[B23] Pleša J. Kozinc Ž. Šarabon N. (2021). The association between force-velocity relationship in countermovement jump and sprint with approach jump, linear acceleration and change of direction ability in volleyball players. Front. Physiol. 12, 763711. doi: 10.3389/fphys.2021.763711 34867467 PMC8637321

[B24] Ramirez-Campillo R. Andrade D. C. Nikolaidis P. T. Moran J. Clemente F. M. Chaabene H. . (2020). Effects of plyometric jump training on vertical jump height of volleyball players: a systematic review with meta-analysis of randomized-controlled trials. J. Sports. Sci. Med. 19, 489–499. PMC7429440 32874101

[B25] Ramirez-Campillo R. García-de-Alcaraz A. Chaabene H. Moran J. Negra Y. Granacher U. (2021). Effects of plyometric jump training on physical fitness in amateur and professional volleyball: a meta-analysis. Front. Physiol. 12, 636140. doi: 10.3389/fphys.2021.636140 33716784 PMC7952872

[B26] Rebelo A. Pereira J. R. Nakamura F. Y. Valente-dos-Santos J. (2025). Beyond the jump: a scoping review of external training load metrics in volleyball. Sports. Health 17, 111–125. doi: 10.1177/19417381241237738 38556860 PMC11569689

[B27] Sattler T. Hadžić V. Dervišević E. Marković G. (2015). Vertical jump performance of professional male and female volleyball players: effects of playing position and competition level. J. Strength. Cond. Res. 29, 1486–1493. doi: 10.1519/JSC.0000000000000781 25436623

[B28] Sheppard J. M. Gabbett T. J. Stanganelli L. C. R. (2009). An analysis of playing positions in elite men’s volleyball: considerations for competition demands and physiologic characteristics. J. Strength. Cond. Res. 23, 1858–1866. doi: 10.1519/JSC.0b013e3181b45c6a 19675472

[B29] Silva A. F. Clemente F. M. Lima R. Nikolaidis P. T. Rosemann T. Knechtle B. (2019). The effect of plyometric training in volleyball players: a systematic review. Int. J. Environ. Res. Public Health 16, 2960. doi: 10.3390/ijerph16162960 31426481 PMC6720263

[B30] Skazalski C. Whiteley R. Bahr R. (2018). High jump demands in professional volleyball—large variability exists between players and player positions. Scand. J. Med. Sci. Sports. 28, 2293–2298. doi: 10.1111/sms.13255 29956376

[B31] Sorenson S. C. Arya S. Souza R. B. Pollard C. D. Salem G. J. Kulig K. (2010). Knee extensor dynamics in the volleyball approach jump: the influence of patellar tendinopathy. J. Orthop. Sports. Phys. Ther. 40, 568–576. doi: 10.2519/jospt.2010.3313 20508329

[B32] Tai W. H. Peng H. T. Song C. Y. Lin J. Z. Yu H. B. Wang L. I. (2021). Dynamic characteristics of approach spike jump tasks in male volleyball players. Appl. Sci. 11, 2710. doi: 10.3390/app1106271030654563

[B33] Yang F. Lu C. Yun X. Qian C. (2026). Effects of neuromuscular training on stability in volleyball athletes: a systematic review and meta-analysis. Front. Sports. Act. Living. 7, 1724934. doi: 10.3389/fspor.2025.1724934 41602798 PMC12832796

[B34] Zahradnik D. Jandacka D. Farana R. Uchytil J. Hamill J. (2014). Ground reaction force and valgus knee loading during landing after a block in female volleyball players. J. Hum. Kinet. 40, 67–75. doi: 10.2478/hukin-2014-0008 25031674 PMC4096083

